# Aggregation-prone A53T mutant of α-synuclein exaggerates methamphetamine neurotoxicity in SH-SY5Y cells: Protective role of cellular cholesterol

**DOI:** 10.1016/j.toxrep.2022.11.006

**Published:** 2022-11-21

**Authors:** Sho Aoki, Takeshi Funakoshi, Toshihiko Aki, Koichi Uemura

**Affiliations:** Department of Forensic Medicine, Graduate School of Medical and Dental Sciences, Tokyo Medical and Dental University, Tokyo, Japan

**Keywords:** Methamphetamine, α-synuclein, Cholesterol, Neurotoxicity, SH-SY5Y cells

## Abstract

The aim of this study is to examine the effects of wild type as well as a mutant (A53T) form of α-synuclein (α-syn) on neuronal cells exposed to methamphetamine (METH). SH-SY5Y human dopaminergic neuronal cells stably expressing exogenously added wild type (WT) or A53T α-syn were established for this purpose. Among the three types of cells, parental, WT α-syn-overexpressing, and A53T α-syn overexpressing SH-SY5Y cells (hereafter referred to as SH-SY5Y, WT SH-SY5Y, and A53T SH-SY5Y, respectively), only A53T SH-SY5Y cells showed significant loss of cell viability after exposure to 2 mM METH for 24 h. Transcriptome analysis using DNA microarray showed that METH induced genes for cholesterol biosynthesis in all of these three cell lines, suggesting that METH upregulates cellular cholesterol biosynthesis independently from cellular α-syn levels. Visualization of the cellular localization of free cholesterol showed that METH induces an aberrant intracellular accumulation of free cholesterol in all three cell lines. In addition, we observed the aggregation of α-syn into cytoplasmic granules, which was more apparent with A53T α-syn than WT α-syn, in cells exposed to METH. Furthermore, the cell death observed in METH-treated A53T SH-SY5Y cells was exaggerated by the addition of 2-hydroxypropyl-β-cyclodextrin (CD), a substance used to extract cholesterol from cells. These results suggest that the aggregation of A53T α-syn in METH-treated cells should be involved in cell death. The upregulation of cellular biosynthesis and cholesterol accumulation by METH should play a protective role against A53T α-syn neurotoxicity in METH-treated SH-SY5Y cells.

## Highlights

• METH induced A53T α-synuclein aggregation in neuronal cells.
• METH also induced cholesterol biosynthesis.
• Cholesterol accumulation ameliorated A53T α-synuclein neurotoxicity.

## Graphical Abstract

**Figure:**
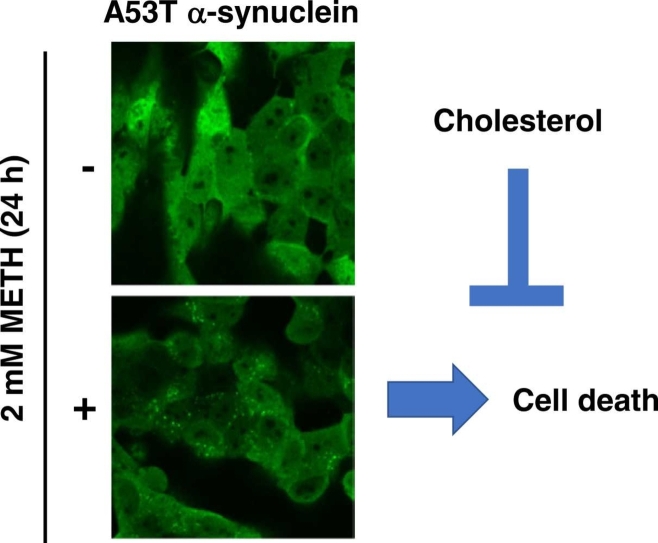

## 1 Introduction

Methamphetamine (METH) is an amphetamine-type psychostimulatory drug abused worldwide. METH abuse in the U.S. at one time declined due to legislation adopted in 2012 that restricted the availability of pseudoephedrine, a compound used to synthesis methamphetamine [1]. However, under the veil of increased public health concerns towards the abuse of opioids, it has been reported that the seizure of methamphetamine produced in Mexico has increased in the U.S in recent years [1], [2]. The effects of METH on humans include euphoric feeling, elevated mood, and elimination of fatigue, and these effects are mainly due to the upregulation of dopamine levels in the dopaminergic neuronal terminus [3]. However, these effects occur only transiently, and the repeated administration of METH leads to a loss of dopaminergic neurons [3], [4], [5]. In accordance with the effects on the human body, there have been many reports demonstrating that METH induces neuronal apoptosis in vitro [6], [7], [8]. METH damages dopaminergic neurons in a variety of ways including through ER stress and mitochondrial respiratory chain dysfunction, both of which seem to be involved in the subsequent apoptotic death of neurons [6].

α-Synuclein (α-syn) is a protein expressed mainly in the central nervous system (CNS) [9]. α-Syn is considered to be involved in the trafficking of neurotransmitters by facilitating SNARE complex formation and the subsequent fusion of vesicles required for the release of neurotransmitters, including dopamine, at pre-synaptic nerve terminals [10]. α-Syn is highly susceptible to cellular stresses and tends to form aggregates that are frequently observed as Lewy bodies (LB) in the brains of patients suffering from dementia or Parkinson’s disease (PD) [11]. Indeed, several mutations in the α-syn gene have been found in families suffering from hereditary PD [12]. A53T α-syn, in which the alanine at residue 53 is replaced by threonine, is one of these hereditary PD-responsible mutant forms of α-syn [13]. One reason for the involvement of A53T α-syn in the onset and/or progression of PD is its increased tendency to aggregate as compared to wild type (WT) α-syn [13].

It has been shown that α-syn interacts with synaptic vesicles by binding to cholesterol in the membrane [14]. Although it has been shown that α-syn aggregation is crucially affected by the levels of cellular cholesterol, there is a serious contradiction about the effect of cholesterol on α-syn aggregation: both higher and lower levels of cholesterol are reported to facilitate α-syn aggregation as well as PD pathogenesis [15], [16], [17]. Recently, Mahapatra et al. suggested that there is an optimal level of cholesterol in synaptic vesicle membranes to facilitate α-syn aggregation, providing a possible explanation for the many contradicting reports regarding the role of cholesterol levels in α-syn aggregation [18].

We found that METH induces cholesterol biosynthesis pathway genes, as we have also observed in cells treated with another drug, L-norephedrine, which is less potent as a psychostimulant than amphetamine or METH [19]. Using stable cell lines expressing WT or A53T α-syn in SH-SY5Y cells, we found that the A53T α-syn toxicity on neuronal cells can be accelerated by a cholesterol extracting agent. Thus, the accumulation of cholesterol in METH-treated cells should contribute to the amelioration of A53T α-syn toxicity, providing further evidence for the critical role of cholesterol in α-syn neurotoxicity.

## 2 Materials and methods

### 2.1 Cell cultures and transfection

SH-SY5Y human neuroblastoma cells, obtained from American Type Culture Collection (ATCC), were maintained as described previously [19]. To create stable cell lines, SH-SY5Y cells were transfected with human WT or A53T α-syn expression vectors using Lipofectamine2000 (Life Technology). Selection of stably transfected cells were performed with the medium containing 400 μg/ml G418 Sulfate (Calbiochem). After selection, WT and A53T SH-SY5Y cells were maintained in the medium containing 200 μg/ml and 300 μg/ml G418, respectively.

### 2.2 DNA microarray analysis

DNA microarray analysis was performed as described previously [19], except that a ClariomS array (Thermo Fisher Scientific) was used in this study.

### 2.3 Measurements of cell viabilities and LDH

Cell viability and LDH release rate were evaluated by Cell Counting Kit-8 (CCK-8) (Dojindo, Kumamoto, Japan) and LDH-Cytotoxic Test *Wako* (FUJIFILM Wako Chemical Corporation, Osaka, Japan), respectively.

### 2.4 Real-time RT PCR

Real-time reverse transcriptase-mediated PCR was conducted as described previously [19]. The primers used are listed in Supplementary Table 1.

### 2.5 Measurement of cholesterol

The amounts of total cellular cholesterol were determined by gas chromatography mass spectrometry (GC-MS) as described previously [19], except a GCMS-TQ8030 (Shimadzu, Kyoto, Japan) and a DB-5 column (30 m long, 0.25-mm inner diameter, 0.25-μm film thickness, Agilent J&W) were used in this study.

### 2.6 Immunostaining and FilipinIII staining

Immunocytochemical analysis was performed as described previously [19]. In brief, cells grown on glass coverslips were treated with or without METH (2 mM) for 6 h and incubated overnight with anti-α-syn antibody (610786; BD Biosciences, New Jersey, USA) and DAPI at 4 ℃. For staining of cellular free cholesterol with of FilipinIII, cells were fixed in 4% paraformaldehyde, and incubated with 75 µg/ml FilipinIII for 30 min. A confocal microscope (C2 +; Nikon, Tokyo, Japan) was used for evaluation of cellular localizations of α-syn as well as free cholesterol.

### 2.7 Immunoblotting

Immunoblotting was carried out as described previously [19]. Antibodies used were as follows; α-syn (610786; BD Biosciences), β-actin (A2066, Sigma), cleaved-caspase3 (#9661, Cell Signaling Technology, CA, USA), GPX4 (ab125066, abcam). LC3 (#2775, Cell Signaling Technology) and peroxidase-conjugated anti-rabbit-IgG and -mouse-IgG antibodies (W4011 and W44021, Promega, Madison, MI).

### 2.8 Statistical analysis

Data are expressed as the mean ± S.D. of at least three independent samples. The data were analyzed by the Tukey-Kramer, Dunnett or Student *t*-tests. *p* values less than 0.05 were considered to be statistically significant.

## 3 Results

### 3.1 Generation of stable cell lines expressing α-syn and the effect of α-syn on METH cytotoxicity

**Fig. 1:**
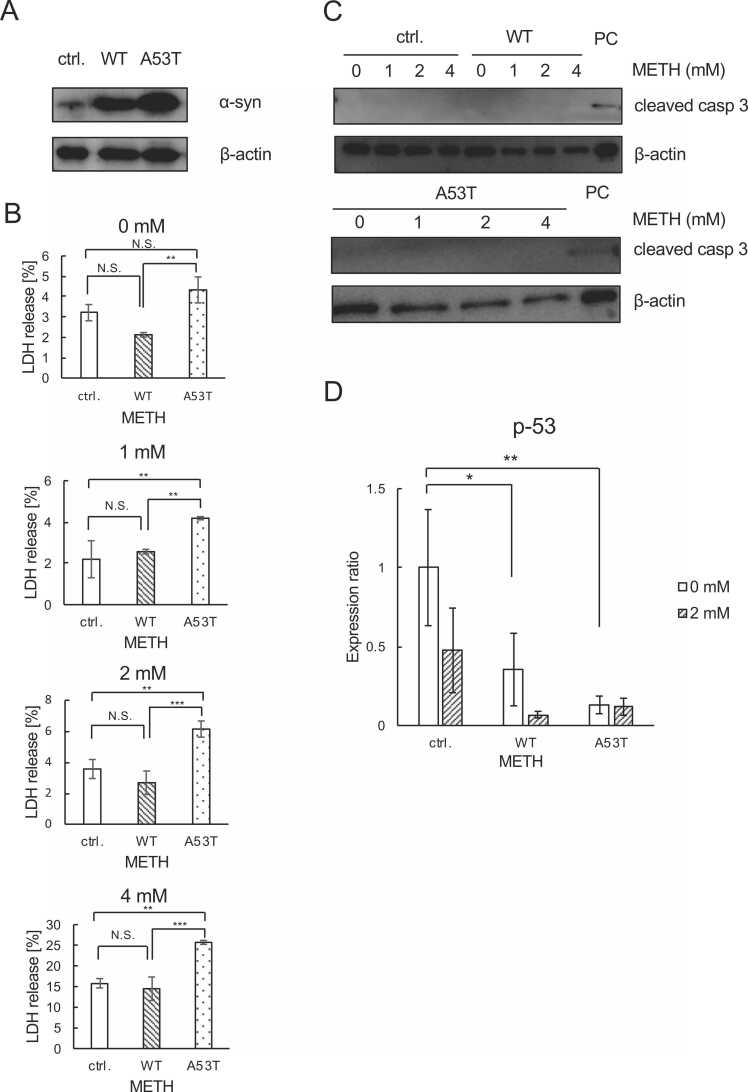
**METH induces non-apoptotic cell death of SH-SY5Y cells.** (A) Establishment of cell lines stably expressing WT and A53T α-syn in SH-SY5Y cells. Immunoblot analysis of α-syn in parental SH-SY5Y cells used as control (ctrl.), as well as WT α-syn-overexpressing (WT) and A53T α-syn overexpressing (A53T) SH-SY5Y cells. (B) Cytotoxicity of METH evaluated by LDH release assay. Cells were treated with the indicated concentrations of METH for 24 h. (C) Immunoblot analysis of cleaved caspase 3 after 24 h exposure to METH. (D) Real time RT-PCR analysis of p-53 mRNA. Cells were treated with the indicated concentrations of METH for 6 h. Tukey-Kramer analysis was used to evaluate statistical significance (p < 0.05, *; p < 0.01, **; p < 0.001, ***).

To investigate the effects of α-syn on the cytotoxicity of METH on SH-SY5Y neuroblastoma cells, we created stable cell lines expressing WT or A53T mutated α-syn (A53T) through transfection of expression vectors for these genes. After selection of SH-SY5Y cells expressing these genes by antibiotics (G418), we confirmed the expression of α-syn in the cells by immunoblotting (Fig. 1A). Clear expression of α-syn was observed in both the WT and A53T SH-SY5Y cells, suggesting the successful generation of stable cell lines expressing WT or A53T α-syn. Next, we investigated the effects of WT as well as A53T α-syn on the cytotoxicity of METH in these cells. Parental SH-SY5Y cells as well as WT and A53T SH-SY5Y cells were treated with or without METH (1, 2 and 4 mM) for 24 h, and cytotoxicity was evaluated by LDH release assay. As shown in Fig. 1B, higher rates of cell death were observed in A53T SH-SY5Y cells as compared with WT or parental SH-SY5Y cells after exposure to 1–4 mM METH for 24 h. Thus, A53T α-syn seems to be more effective than WT α-syn in enhancing cell death by METH. To investigate whether METH-induced cell death is apoptosis or not, we examined cleaved caspase3 by immunoblotting. As shown in Fig. 1C, no cleaved caspase3 was observed at any concentration of METH treatment in any of the parental, WT, or A53T SH-SY5Y cells. In accordance with the lack of apoptosis in METH-treated SH-SY5Y cells, the expression of p-53 was rather decreased by METH (Fig. 1D). Collectively, these results show that the overexpression of A53T α-syn exaggerates METH-induced SH-SY5Y cell death, and that this cell death is distinct from apoptosis.

### 3.2 METH induces autophagy but not ferroptosis

**Fig. 2:**
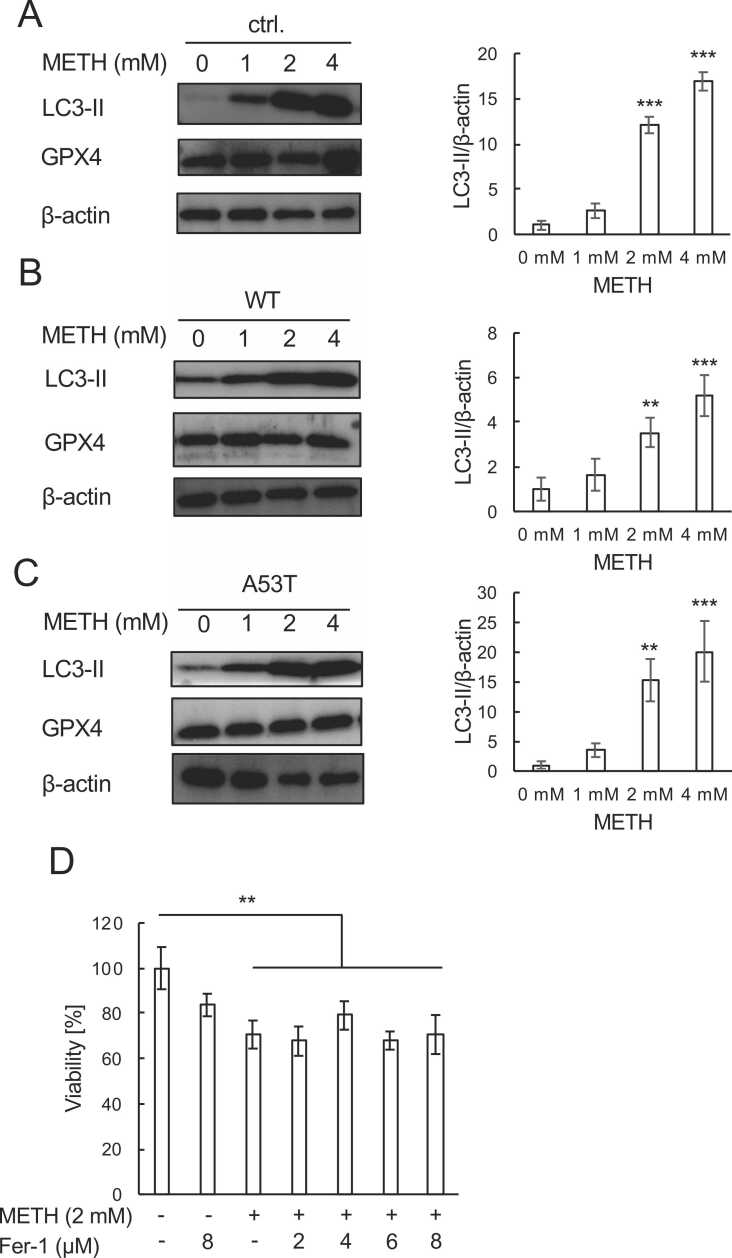
**METH induces autophagy but not ferroptosis in SH-SY5Y cells.** (A-C) Effects of METH on the status of markers of autophagy (LC3-II) and ferroptosis (GPX4). Control (ctrl.), as well as WT α-syn-overexpressing (WT) and A53T α-syn overexpressing (A53T) SH-SY5Y cells were treated with 1, 2, or 4 mM METH for 24 h followed by immunoblot analysis to evaluate LC3-II and GPX4 levels. β-actin served as a loading control. Dunnett’s test was used to evaluate statistical significance (p < 0.01, **; p < 0.001, ***). (D) Effects of the ferroptosis inhibitor ferrostatin (fer-1) on cell death induced by METH. Cells were treated with the indicated concentrations of METH and fer-1 for 24 h, and MTT assays were performed to evaluate cell viabilities. Tukey-Kramer analysis was used to evaluate statistical significance (p < 0.01, **).

To obtain further information about the mode of SH-SY5Y cell death by METH, we examined LC3-II and GPX4, marker proteins for autophagy and ferroptosis, respectively, in these cells. Parental as well as WT and A53T cells were treated with or without METH (1, 2 and 4 mM) for 24 h and examined by immunoblotting. As shown in Fig. 2A-C, the expression of LC3-II was significantly increased by METH in a concentration-dependent manner in all of these cells, suggesting the induction of autophagy by METH (Fig. 2A-C). On the other hand, the levels of GPX4 showed no changes with or without METH, indicating that ferroptosis is not involved in cell death by METH. Finally, we checked the effects of ferrostatin-1 (2, 4, 6 and 8 μM) on METH-induced cell death to confirm further that ferroptosis is not involved in the METH-induced death of A53T cells. As shown in Fig. 2D, ferrostatin-1 did not affect the loss of viability of cells treated with METH. These results suggest that the METH-induced death of A53T cells involves autophagy but not ferroptosis.

### 3.3 METH induces cholesterol synthesis pathway genes

**Fig. 3:**
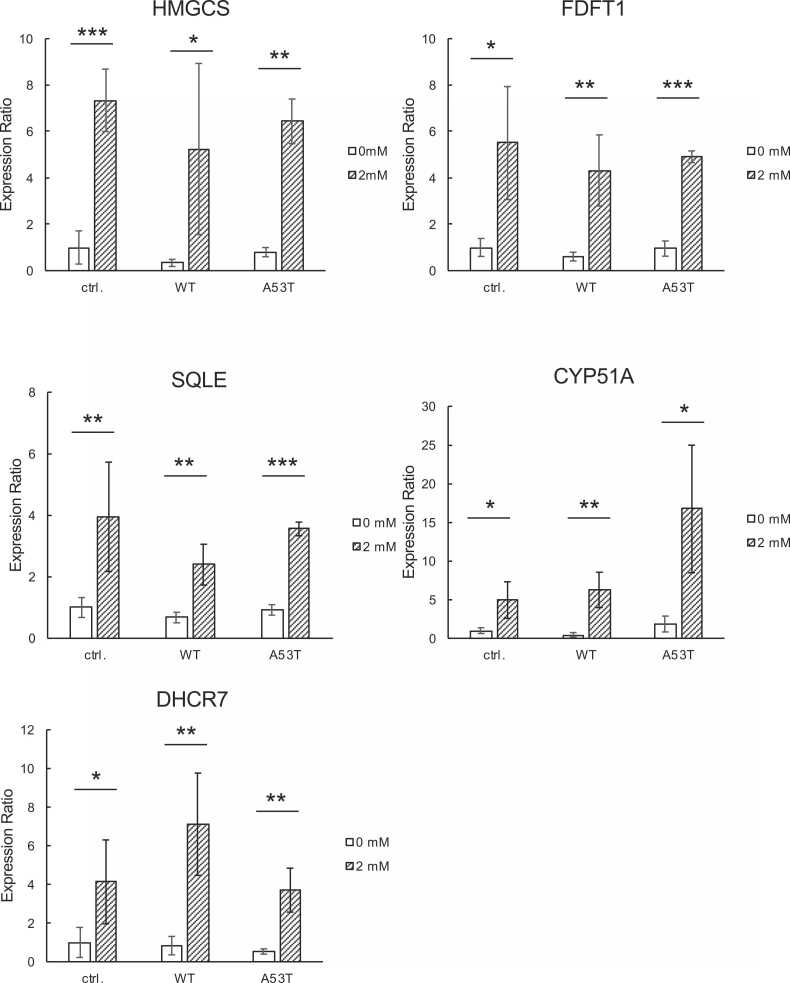
**METH induces the expressions of genes for cholesterol biosynthesis.** Control (ctrl.), as well as WT α-syn-overexpressing (WT) and A53T α-syn overexpressing (A53T) SH-SY5Y cells were treated with 2 mM METH for 6 h, and real time RT-PCR analysis was conducted to evaluate the expressions of the genes for 3-hydoroxy-3-methyglutaryl-Coenzyme A synthase 1 (HMGCS), farnesyl-diphosphate farnesyltransferase 1 (FDFT1), squalene epoxidase (SQLE), lanosterol 14α-demethylase (CYP51A), and 7-dehydrocholesterol reductase (DHCR7). Student’s t test was used to evaluate statistical significance (p < 0.05, *; p < 0.01, **; p < 0.001, ***).

To investigate further the molecular mechanisms underlying METH-induced cell death, we investigated changes in gene expression caused by treatment with METH (2 mM) for 6 h using a DNA microarray technique. Table 1 shows the top 10 genes that showed increased expressions by METH, many of which are involved in the biosynthesis of cholesterol. Among them we chose 3-hydoroxy-3-methyglutaryl-Coenzyme A synthase 1 (HMGCS), farnesyl-diphosphate farnesyltransferase 1 (FDFT1), squalene epoxidase (SQLE), lanosterol 14α-demethylase (CYP51A), and 7-dehydrocholesterol reductase (DHCR7), and confirmed their gene expressions by reverse transcriptome-mediated real-time PCR. All of these genes showed significantly increased expression upon treatment with METH (Fig. 3). These results suggest that the cholesterol synthesis pathway is induced by METH.

**Table 1:** TOP10 genes showing increased expressions in response to METH treatment (2 mM, 6 h) in parental, WT, and A53T SH-SY5Y cells.

**Parental SH-SY5Y cells**			
Gene symbol	RefSeq Transcript ID	Fold change	Gene name
HMGCS1	NM_001098272	7.51	3-hydroxy-3-methylglutaryl-CoA synthase 1 (soluble)
CYP51A1	NM_000786	5.13	cytochrome P450, family 51, subfamily A, polypeptide 1
LINC00473	NR_026860	4.95	long intergenic non-protein coding RNA 473
INSIG1	NM_005542	4.63	insulin induced gene 1
MSMO1	NM_001017369	4.58	methylsterol monooxygenase 1
DHCR7	NM_001163817	4.49	7-dehydrocholesterol reductase
UTRN	NM_007124	3.72	utrophin
DHCR24	NM_014762	3.58	24-dehydrocholesterol reductase
FDFT1	NM_001287742	3.44	farnesyl-diphosphate farnesyltransferase 1
STARD4	NM_001308056	3.39	StAR-related lipid transfer domain containing 4
**WT SH-SY5Y cells**			
LINC00473	NR_026860	7.30	long intergenic non-protein coding RNA 473
HMGCS1	NM_001098272	7.13	3-hydroxy-3-methylglutaryl-CoA synthase 1 (soluble)
MSMO1	NM_001017369	5.98	methylsterol monooxygenase 1
DHCR7	NM_001163817	5.64	7-dehydrocholesterol reductase
INSIG1	NM_005542	5.20	insulin induced gene 1
LDLR	NM_000527	4.88	low density lipoprotein receptor
FDFT1	NM_001287742	4.61	farnesyl-diphosphate farnesyltransferase 1
UTRN	NM_007124	4.26	utrophin
STX3	NM_001178040	4.23	syntaxin 3
HSD17B7	NM_001304512	4.10	hydroxysteroid (17-beta) dehydrogenase 7
**A53T SH-SY5Y cells**			
HMGCS1	NM_001098272	7.72934	3-hydroxy-3-methylglutaryl-CoA synthase 1 (soluble)
INSIG1	NM_005542	5.80464	insulin induced gene 1
MSMO1	NM_001017369	5.43597	methylsterol monooxygenase 1
LDLR	NM_000527	5.16705	low density lipoprotein receptor
DHCR7	NM_001163817	4.87366	7-dehydrocholesterol reductase
FBXO8	(Entrez Gene ID 26269)	4.66319	F-box protein-8
LINC00473	NR_026860	4.30869	long intergenic non-protein coding RNA 473
BIRC6	(Entrez Gene ID 57448)	3.81363	Baculoviral IAP repeat containing 6
DHCR24	NM_014762	3.62984	24-dehydrocholesterol reductase
CYP51A1	NM_000786	3.61332	cytochrome P450, family 51, subfamily A, polypeptide 1

### 3.4 METH does not increase cellular cholesterol levels

**Fig. 4:**
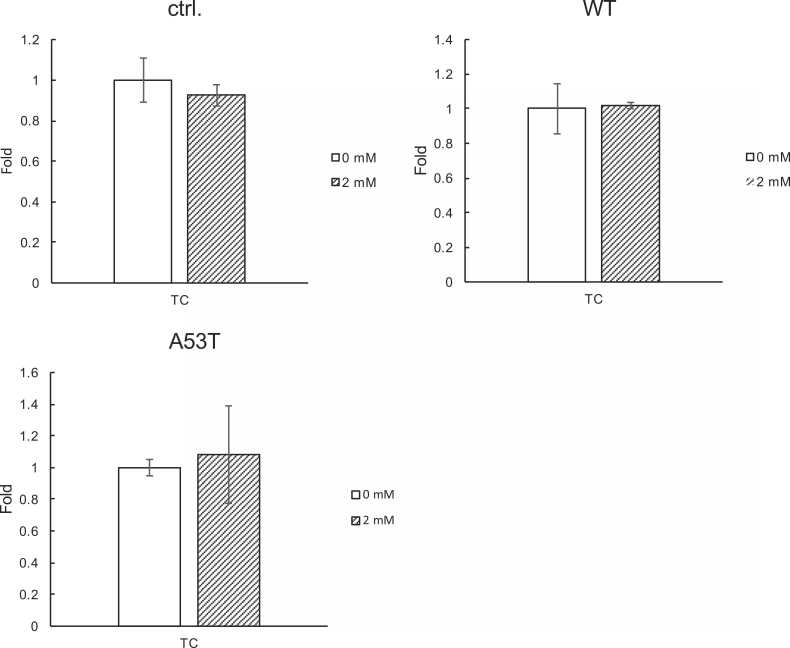
**METH has no effects on cellular cholesterol levels.** Control (ctrl.), as well as WT α-syn-overexpressing (WT) and A53T α-syn overexpressing (A53T) SH-SY5Y cells were treated with 2 mM METH for 6 h, and total cholesterol levels were measured by GC-MS.

Given the indication of the increased expressions of the genes for cholesterol biosynthesis (Fig. 3), we examined cholesterol levels in cells treated with or without METH by gas chromatography mass spectrometry (GC-MS). We observed no changes in cellular cholesterol levels in any of the parental, WT, or A53T SH-SY5Y cells between control and METH-treated cells (Fig. 4).

### 3.5 Intracellular aggregation of α-syn in METH-treated SH-SY5Y cells

**Fig. 5:**
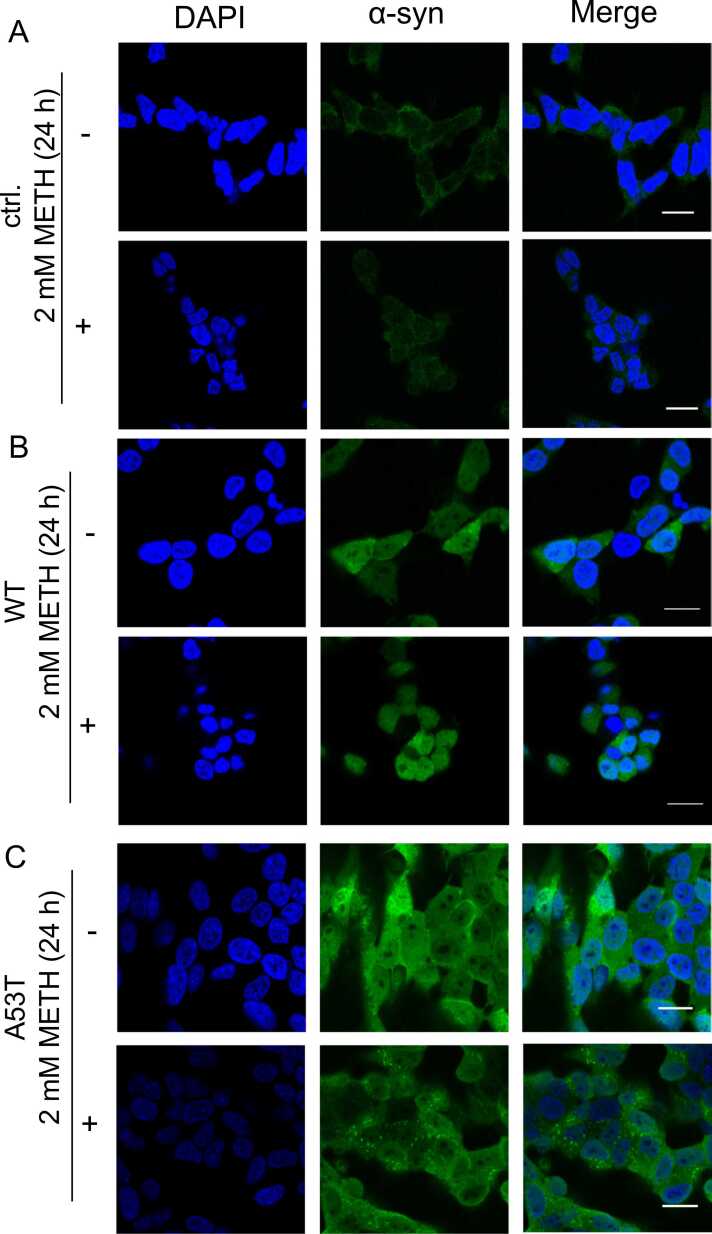
**METH induces α-syn aggregation in SH-SY5Y cells.** Control (ctrl.), as well as WT α-syn-overexpressing (WT) and A53T α-syn overexpressing (A53T) SH-SY5Y cells were treated with 2 mM METH for 24 h, and immunocytochemical analysis was conducted to evaluate the cellular localization of α-syn. The cells were incubated with anti-α-syn antibody (green), and nuclei were also stained with DAPI (blue) and observed by fluorescence microscopy. Scale bar, 50 µm. (For interpretation of the references to colour in this figure legend, the reader is referred to the web version of this article.)

We next examined the cellular localization of α-syn in cells with or without METH treatment (2 mM, 24 h) by immunofluorescence microscopy. Parental SH-SY5Y cells showed almost no staining with the anti-α-syn antibody (Fig. 5A), consistent with the immunoblotting results indicating a lack of α-syn expression in SH-SY5Y cells (Fig. 1A). In WT cells treated with and without METH, granular localization and/or aggregation of α-syn was occasionally observed (Fig. 5B). In A53T cells, more granules were observed after METH treatment as compared with WT cells (Fig. 5C). These results indicate that METH facilitates A53T α-syn aggregation, which could be involved in the cell death caused by METH.

### 3.6 Localization of intracellular cholesterol and the effects of 2-hydroxypropyl-β-cyclodextrin

**Fig. 6:**
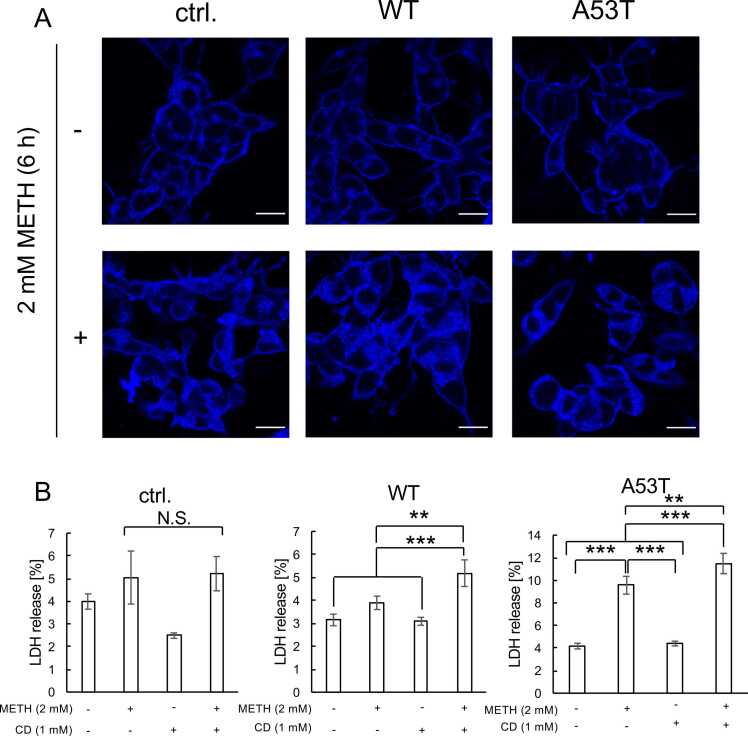
**METH induces the cellular accumulation of free cholesterol in SH-SY5Y cells.** (A) FilipinIII staining. Control (ctrl.), as well as WT α-syn-overexpressing (WT) and A53T α-syn overexpressing (A53T) SH-SY5Y cells were treated with 2 mM METH for 6 h, followed by staining with FilipinIII, and observation under a fluorescence microscope to visualize cellular free cholesterol. Scale bar, 50 µm. (B) Effects of 2-hydroxypropyl-β-cyclodextrin (CD) on METH-induced cell death. Cells were treated 2 mM METH for 24 h, and MTT assays were performed to evaluate cell viability. Tukey-Kramer analysis was used to evaluate statistical significance (p < 0.05, *; p < 0.01, **; p < 0.001, ***).

Finally, we examined the intracellular localization of free cholesterol using Filipin III, a fluorescence dye that selectively stains the non-esterified free form of cholesterol. As shown in Fig. 6A, free cholesterol was mainly localized at the plasma membrane in non-METH treated parental, WT and A53T cells. In contrast, METH induces the intracellular accumulation of free cholesterol in all three cells (Fig. 6A). It should be noted that the fluorescence intensities in METH-treated cells were apparently stronger than in non-treated cells in all parental, WT and A53T cells (Fig. 6A). Thus, METH seemed to upregulate the levels of intracellular free cholesterol. We further examined whether the intracellular accumulation of free cholesterol has any adverse effects on the cells. For this, we examined the effects of 2-hydroxypropyl-β-cyclodextrin (CD), which promotes the release of cholesterol from cells, on the cell death caused by METH. Treatment with CD (1 mM) exaggerated the METH-induced death in WT and A53T cells, indicating that the intracellular accumulation of cholesterol should have a protective effect on METH toxicity in cells harboring α-syn.

## 4 Discussion

In this study, we examined whether or not α-syn aggravates METH neurotoxicity. Our results show that A53T α-syn, which undergoes aggregation in response to 2 mM METH exposure (Fig. 5C), accelerates METH-induced death of SH-SY5Y neuronal cells. Interestingly, cellular cholesterol seems to alleviate α-syn neurotoxicity in both WT and A53T cells since cell death was enhanced by CD, a cholesterol extracting agent (Fig. 6B).

In the vast majority of multicellular organisms, apoptosis plays a crucial role in the establishment and maintenance of homeostasis. The apoptotic process involves biological and structural changes to the mitochondria, including mitochondrial dysfunction, disruption of the outer membrane of the mitochondria, and the release of cytochrome c, which may cause Apaf1 and caspase9 to connect, activating caspase9. Then, caspase9 activates caspase3, which causes cellular and morphological abnormalities and ultimately leads to cellular death. A crucial role for mitochondria in mediating the apoptotic process has been confirmed in various types of chemical toxicity such as sodium fluoride as well as vancomycin [20], [21].

Our results indicate that A53T α-syn can potentiate METH neurotoxicity (Fig. 1B), suggesting that the mechanism of neurotoxicity of METH and A53T α-syn should be, at least in parts, the same. Other than the formation of LB, PD is characterized by the loss of dopamine neurons. METH alters dopamine metabolism and leads to subsequent loss of dopamine neurons [4]. In its normal context, α-syn facilitates the membrane trafficking system involved in the dopamine metabolism [22]. Since aggregation-prone A53T α-syn has been reported to interrupt dopamine signaling [23], it might be possible that both METH and A53T α-syn target dopamine metabolism and/or signaling, which finally leads to the death of SH-SY5Y cells.

Although many reports have indicated crucial roles of mitochondrial apoptosis in METH neurotoxicity [24], we observed no caspase3 cleavage in cells treated with METH (Fig. 1C). This result is consistent to our previous report [25]. Ferroptosis also seems not to be involved in cell death in response to METH (Fig. 2). Our previous results showed necroptosis in SH-SY5Y cells treated with L-norephedrine, which shares a structural similarity with METH [19]. Indeed, Kun et al. demonstrated the involvement of necroptosis in METH neurotoxicity in embryonic rat cortical neurons [26]. Although necroptosis is highly suspected to be responsible for the neurotoxicity observed in our current study, further investigation is needed to clarify the mode of cell death caused by METH in our experimental settings.

In accordance with our current results that METH-induced SH-SY5Y cell death is not apoptosis (Fig. 1C), we did not observe any increase of p-53 transcription; we did observe the decrease of it (Fig. 1D). Interestingly, direct transactivation of α-syn gene expression by p-53 has been reported [27]. Significant decrease of p-53 expression in WT and A53T cells compared to parental cells (Fig. 1D) might indicate that there is a negative feedback loop between α-syn and p-53 expression levels.

Autophagy contributes to maintain cellular homeostasis. Microtubule-associated protein light chains 3 (LC3), a mammalian autophagy protein, is a sign of autophagosomes. The most often employed marker is LC3B, one of the four LC3 isoforms [28]. We observed significant up regulation of the active form of LC3 (LC3-II) in response to METH exposure (Fig. 2 A-C). Thus, autophagy might contribute to maintain cellular homeostasis in METH-treated SH-SY5Y cells.

We also observed the induction of cholesterol biosynthesis genes by METH in parental, WT, and A53T SH-SY5Y cells (Fig. 3 and Table 1). Our results also indicate unchanged levels of total cholesterol (Fig. 4) and increased levels of free cholesterol (Fig. 6A) in METH-treated cells as compared with untreated cells. This suggests that an esterified form of cholesterol is somewhat decreased in METH-treated cells as compared with untreated cells. In general, the accumulation of non-esterified free cholesterol should have bad effects on cells [29]. Excessive free cholesterol is removed from cell membranes and transported to the ER where free cholesterol is esterified by acyl-CoA cholesterol acyltransferase and subsequently stored as lipid droplets [30]. Therefore, the induction of the cholesterol biosynthesis pathway by METH might result in the accumulation of free cholesterol in cellular membranes instead of being properly stored as lipid droplets. However, the increased levels of cellular cholesterol might be involved in protecting cells against the neurotoxicity of α-syn aggregation [18]. Given the result that the extraction of cellular cholesterol by CD resulted in the acceleration of METH cytotoxicity in A53T SH-SY5Y cells (Fig. 6B), it appears rational to conclude that cholesterol is involved in the protection against METH neurotoxicity, at least in A53T SH-SY5Y cells in some experimental settings.

In conclusion, we demonstrate that α-syn facilitates METH toxicity in neuronal cells. We also show that cellular cholesterol appears to play a protective role against neurotoxicity caused by the combination of A53T α-syn and METH. These findings may shed light on the importance of cholesterol homeostasis in protecting cells against the neurotoxicity of several neurotoxic substances and proteins, such as METH and α-syn.

## Funding

This study was supported by a grant-in-aid from MEXT KAKENHI (grant number 19K10682 to T.F. and 18K19670 to T.A.).

## CRediT authorship contribution statement

**Sho Aoki**: Investigation, Validation, Data curation, Writing – original draft, Visualization. **Takeshi Funakoshi**: Conceptualization, Data curation, Project administration, Formal analysis, Funding acquisition**. Toshihiko Aki**: Project administration, Funding acquisition, Writing – review & editing. **Koichi Uemura**: Supervision, Project administration.

## Declaration of Competing Interest

The authors declare that they have no known competing financial interests or personal relationships that could have appeared to influence the work reported in this paper.

## Data Availability

Data will be made available on request.
