# Supplementary material for: Aggregation-prone A53T mutant of α-synuclein exaggerates methamphetamine neurotoxicity in SH-SY5Y cells: Protective role of cellular cholesterol
Source: Toxicol Rep. 2022 Nov 21;9:2020–9. doi: 10.1016/j.toxrep.2022.11.006 (PMC9742969; doi:10.1016/j.toxrep.2022.11.006)
Supplement: Supplementary file 1 — Supplementary material [file mmc1.docx]

**Supplementary Table 1**

Sequence of primers used for reverse transcriptase-mediated real time PCR

| Gene | Forward primer (5’-3’) | Reverse primer (5’-3’) |
| --- | --- | --- |
| HMGCS | GATGCTACACCGGGGTCTG | ATCATTTGGAGTGGGACGCC |
| FDFT1 | CCTCGAAGCACCTACTCCAC | AGCGAGTCCTGGTCCATCTT |
| CYP51A | CGACCTCGGCCTTCAGTGTT | ATGGAGGACTTTTCACCCCTG |
| DHCR7 | ACAGAACCGCATCTCAAGGG | ACGTGTACAGAAGCACCTGG |
| SQLE | CTGCCACAGATGATTCCCTG | ACTGAGAAGGGCTCGAGGTT |
| β-actin | CCACGAAACTACCTTCAACTCC | TCATACTCCTGCTGCTTGCTGATCC |
